# Supplementary material for: Work engagement, impulsivity and, self-efficacy among Polish workers. Moderating role of impulsivity
Source: PLoS One. 2024 Oct 10;19(10):e0311856. doi: 10.1371/journal.pone.0311856 (PMC11466399; doi:10.1371/journal.pone.0311856)
Supplement: Attachment — Submitted filename: Rev..docx [file pone.0311856.s001.docx]

**Dear authors,**

**I want to express my gratitude for allowing me the opportunity to review your research paper. It's evident that you've put considerable effort into your work, and I commend your dedication to advancing the field.**

**After a thorough review, I'd like to provide you with some feedback and recommendations to enhance the overall quality of your paper. I understand the importance of constructive feedback, and I believe these suggestions could strengthen your work and increase its chances of successful publication.**

**General Feedback**

**1. Clarity and Structure:**

Certain sections of your paper could benefit from improved clarity and concise language. Consider revisiting sentences and ensuring that the flow of ideas is seamless. This will enhance the overall readability of your paper.

**2. Grammar and Language:**

While the paper is generally well-written, there are a few grammatical issues that could be addressed to improve clarity. Be attentive to sentence structure and ensure consistency in verb tenses throughout the document.

**3. Additional Information:**

The paper appears to lack some essential elements that are typically expected in research articles. Including a more detailed literature review, a clear research question or hypothesis, and robust methodology would strengthen the overall framework of your study.

**I believe that incorporating these suggestions will significantly improve the quality of your research paper. I understand the effort and dedication required in academic writing, and I hope you find these recommendations helpful.**

**Detailed review**

**Abstract:**

*Clarity and Conciseness:*

The abstract is generally clear in presenting the study's purpose, methodology, and key findings.

However, certain sentences could be streamlined for better clarity and conciseness.

For example,

Original: "The study took up the idea of adding a new component to the Job Demands - Resources Theory (Bakker, Demerouti 12 & Sanz-Vergel, 2023), which should be called “personal demands”."

Revised: "The study proposes adding a new component to the Job Demands-Resources Theory, termed 'personal demands'."

Original: "As an example of personal demands, the construct of Impulsivity was studied."

Revised: "Impulsivity serves as an illustrative example of personal demands."

*Statistical Information:*

The abstract mentions a negative correlation between impulsivity and work engagement but does not provide effect sizes or statistical significance levels. Including such information would strengthen the credibility of the findings.

The citation for Bakker, Demerouti, and Sanz-Vergel (2023) should be removed from the abstract.

**Introduction:**

1- The introduction provides multiple perspectives on the definition of work engagement, particularly referring to Kahn's conceptualization. Consider consolidating these perspectives into a concise and cohesive definition for clarity.
2- The discussion about work engagement being a temporary condition is important. Consider providing a brief example or illustration to make this concept more tangible for readers.
3- The passage discusses stable resources and proposes the distinction of organizational and key personal resources. Consider briefly explaining these categories or providing examples to enhance understanding.
4- Ensure consistency in terminology. For instance, the use of "work engagement" and "engagement at work" could be standardized for clarity.
5- Carefully review grammar and syntax to ensure accurate expression of ideas. For example, in sentence 30, "in work engagement" might need clarification for smoother understanding.
6- The authors effectively highlight the research gap related to personal demands within the JD-R model. The purpose of the study is clearly stated as investigating the impact of impulsivity on motivational states.
7- The inclusion of prior research on impulsivity and its negative consequences, such as drug-taking, eating disorders, and risky behaviors, adds depth to the understanding of impulsivity. However, it might be helpful to briefly connect these findings to the workplace context to establish relevance.
8- The second hypothesis introducing impulsivity as a moderator of the relationship between self-efficacy beliefs and work engagement is logically justified. This connection is consistent with the JD-R model and Bakker and Demeroouti's suggestion.

**Method:**

***Tools***

1- Ensure consistency in the use of terminology. For instance, the Utrecht Work Engagement Scale is mentioned in terms of "Vigour," "Absorbtion," and "Dedication." Consider using standardized terms such as "Vigor," "Absorption," and "Dedication" for consistency.
2- Provide a brief overview of the five subscales of the SUPPS-P Impulsive Behavior Scale (positive UR, negative UR, premeditation, perseverance, and sensation seeking) for a better understanding of the impulsivity construct.

***Procedure***

3- The timeline for the survey is mentioned due to the COVID-19 pandemic, which is relevant. However, it might be beneficial to briefly explain why this specific time frame was chosen or if there were any external factors influencing the decision.
4- The description of contacting potential respondents by phone and sharing the questionnaire through Google Forms due to the pandemic is clear. However, consider briefly explaining how this remote data collection method might influence data quality or participant responses.
5- The informed consent process is described, which is good. However, explicitly mention that participants were informed about the study's purpose, conditions, and their rights. Ensure that the process aligns with ethical standards.
6- Given the circumstances of the pandemic, the decision to avoid direct contact is justified. However, acknowledging this limitation and discussing any potential impact on response rates or data quality would add transparency.
7- While it's mentioned that participants had the ability to withdraw at any stage, consider adding a sentence emphasizing that withdrawal would not result in any negative consequences.
8- Ensure consistency in the presentation of dates. For example, "15-th" could be standardized as "15th" for clarity.

***Sample:***

9- The three provinces (Malopolskie, Podkarpackie, Swietokrzyskie) are mentioned, but it might be helpful to provide a brief context or rationale for choosing these specific regions. Are they representative of a larger population, or were they chosen for a particular reason?
10- The distribution of education levels is detailed, but it might be beneficial to combine the categories for clarity. For example, the group "Bachelor of Arts" and "Master of Arts" together as "University Degrees" to simplify presentation.

**Results**

1- The detailed breakdown of impulsivity scales (lack of premeditation, lack of perseverance, negative urgency, positive urgency, and sensation seeking) is valuable. However, the use of terms like "average" and "above average" may benefit from more specific descriptors or comparisons.
2- The correlation analysis is appropriately presented but consider providing a brief explanation or interpretation of what the correlations signify in terms of the relationships between variables. This can help readers understand the strength and direction of associations.
3- Statistical information, including F-values, degrees of freedom, and p-values, is provided, which is essential. However, consider providing more detail on the coefficients and standard errors for each predictor variable to offer a comprehensive understanding of the results.
4- It's mentioned that the Hayes Model 1 was applied for moderation analysis. In the section of statistical analysis (Missed), consider briefly explaining the key components of the Hayes Process Model 1 for readers unfamiliar with this statistical approach.

**Discussion:**

1- While the discussion provides an overview of the relationships between impulsivity and work engagement, consider a more detailed discussion of each component of impulsivity and its specific impact on work engagement.
2- Ensure consistent use of terminology. For example, in some instances, the term "impulsivity" is used, while in others, it is referred to as "lack of perseverance." Maintain consistency in naming the components.
3- For the significant findings, such as lack of perseverance being a negative predictor of work engagement, provide a more detailed interpretation of these results. Why is lack of perseverance negatively correlated with work engagement?
4- If there are unexpected or contradictory findings, address them explicitly. For instance, why does sensation seeking correlate positively with work engagement while other components of impulsivity show negative correlations?
5- When discussing the moderation effect of positive urgency on the relationship between self-efficacy and work engagement, provide a detailed explanation of the practical implications and potential reasons behind this moderation.
6- Expand on the practical implications of the findings. How can organizations use this information to improve employee engagement or manage personal demands effectively?
7- Please, conclude the discussion by summarizing the key findings, their theoretical and practical implications, and potential avenues for future research.
