# Supplementary material for: Work engagement, impulsivity and, self-efficacy among Polish workers. Moderating role of impulsivity
Source: PLoS One. 2024 Oct 10;19(10):e0311856. doi: 10.1371/journal.pone.0311856 (PMC11466399; doi:10.1371/journal.pone.0311856)
Supplement: Attachment — Submitted filename: renamed_7991a.docx [file pone.0311856.s003.docx]

Dear Editor,

Regarding the general comment about adjudicating relationships between variables, I want to note that I realize that cross-sectional studies do not provide a basis for inferring causal relationships. However, in the case of our studies , which analyze the relationships of variables, we assume the order of the variables in the relationship on the basis of JD-R theory. The values of correlation and regression coefficients confirm the existence of the relationship postulated by the theory and give information on the strength of the relationship. On the other hand, Impulsivity, which is considered in psychology to be a fixed trait determined genetically, cannot be the result of work engagement or belief of self-efficacy.

| Reviewer | Response |
| --- | --- |
| The title should clearly indicate the independent, dependent, and moderating variables, as well as the subject of the study. | The title has been changed. Its first element is the explained variable. Then the explanatory variables and the group on which the study was done are listed. |
| The introduction would greatly benefit from a more comprehensive background on the JD-R theory and its evolution. | The section on JD-R has been revised. The order of inclusion of each term in the theory and their definitions have been marked. |
| A more detailed justification for introducing 'personal demands' within the JD-R framework would strengthen the theoretical foundation. | The definition of Personal demands has been added. Definitions of all categories of resources and requirements established on JD-R grounds have been placed close together. |
| What about tool validity? Do the authors use tools in Polish language or English? A pilot study and Ethical considerations must be added to the text, especially if you collect data online | A Polish version of the scale was used in the study. All the scales used have their method validation publications in the Polish-language version. These publications are referenced in the text. |
| Nothing was mentioned about the sampling technique and sample size calculation | The survey targeted the general working population. The sample size was due to obtaining sufficient test power and the requirements for multivariate regression methods. We were able to collect more people than the required criterion. |
| The discussion could benefit from a more detailed analysis of each impulsivity component and its specific impact on work engagement. This includes explaining why certain components, like sensation seeking, correlate differently | Thank you for this comment. We have detailed each component of impulsivity to show how it correlates with work engagement. |
| Addressing unexpected or contradictory findings in more detail and providing potential explanations would strengthen the discussion  **Future Research Directions:** Outlining potential avenues for future research, including longitudinal studies or experimental designs, would provide a strong closing to the paper | In accordance with the reviewer's suggestions, we supplemented the discussion by also discussing unexpected and contradictory relationships obtained in the study |
| **Summary of Key Findings:** The conclusion could provide a succinct summary of the key findings, their theoretical contributions, and practical implications | The indicated elements to the text have been added. |
| **Consistency in Terminology:** Ensuring consistent use of terminology throughout the paper, especially regarding constructs like 'work engagement' and its components. | Misspelled names of work engagement factors were identified and corrected. |
| **Grammar and Syntax:** A thorough review of grammatical and syntactical errors to ensure clarity and professionalism in writing. | The text was subjected to further proof editing |
| **Reviewr 3.** |  |
| Please define the concept of personal resources. | The definition of personal resources and personal demands was added on p. 3. |
| 2. Please argue why impulsivity could be considered a personal demand from the JD-R perspective.  The authors should try to add the most important literature (2019-2023) to the topic, deeply demonstrate an understanding of the literature, review the literature critically, and point out limitations as well as conflicts. | We also recognize some shortcomings of JD-R Theory. We point out the need to supplement the model with the construct of Impulsivity taking into account the comments from the article critical of JD-R. He wants to point out that we refer mainly to the latest version of the theory published by Bakker et al. 2023.  (Bakker, A. B., Demerouti, E., & Sanz-Vergel, A. (2023). Job demands–resources theory: Ten years later. Annual Review of Organizational Psychology and Organizational Behavior, 10, 25–53. [https://doi.org/10.1146/annurev-orgpsych-120920-053933](https://psycnet.apa.org/doi/10.1146/annurev-orgpsych-120920-053933)) |
| 3. Please separate the discussion section from Conclusion | It has been separated by adding a separate section entitled Conclusion. |
| 4. The theoretical implication section needs to be created and completed. The research's theoretical implication needs to be discussed in relation to the first part of the article's conceptual framework.  I think the discussions could be enriched by focusing on these since it would better guide future research and alternative explanations for the findings. | Thank you for this comment. The theoretical section on personal demands (Impulsivity) has been completed and explained in the discussion section. |
| 6. What are the practical implications of this research? | It was added in Conclusion. |
